# Effectiveness of peer support to increase uptake of retinal examination for diabetic retinopathy: study protocol for the DURE pragmatic cluster randomized clinical trial in Kirinyaga, Kenya

**DOI:** 10.1186/s12889-018-5761-6

**Published:** 2018-07-13

**Authors:** Nyawira Mwangi, Mark Ng’ang’a, Esbon Gakuo, Stephen Gichuhi, David Macleod, Consuela Moorman, Lawrence Muthami, Peter Tum, Atieno Jalango, Kibata Githeko, Michael Gichangi, Joseph Kibachio, Covadonga Bascaran, Allen Foster

**Affiliations:** 1 Kenya Medical Training College, Nairobi, Kenya; 2 International Centre for Eye Health, London School of Hygiene and Tropical Medicine, Keppel Street, WC1E 7HT, London, UK; 3 Kerugoya County Referral Hospital, Kerugoya, Kenya; 4 Kirinyaga County Health Services, Kerugoya, Kenya; 5 Department of Ophthalmology, University of Nairobi, Nairobi, Kenya; 6 Oxford University Hospitals NHS Trust, Oxford, UK; 7 Kenya Medical Research Institute, Nairobi, Kenya; 8 Kabarak University, Nakuru, Kenya; 9 Upper Hill Eye and Laser Centre, Nairobi, Kenya; 10 Ophthalmic Services Unit, Ministry of Health, Nairobi, Kenya; 11 Division of Non-Communicable Diseases, Ministry of Health, Nairobi, Kenya

**Keywords:** Diabetes mellitus, Diabetic retinopathy, Diabetes support groups, Retinal screening, Blindness, Health education, Self-efficacy theory, Peer-support, Kenya, Africa

## Abstract

**Background:**

All patients with diabetes are at risk of developing diabetic retinopathy (DR), a progressive and potentially blinding condition. Early treatment of DR prevents visual impairment and blindness. The natural history of DR is that it is asymptomatic until the advanced stages, thus annual retinal examination is recommended for early detection. Previous studies show that the uptake of regular retinal examination among people living with diabetes (PLWD) is low. In the Uptake of Retinal Examination in Diabetes (DURE) study, we will investigate the effectiveness of a complex intervention delivered within diabetes support groups to increase uptake of retinal examination.

**Methods:**

The DURE study will be a two-arm pragmatic cluster randomized clinical trial in Kirinyaga County, Kenya. Diabetes support groups will be randomly assigned to either the intervention or usual care conditions in a 1:1 ratio. The participants will be 700 PLWD who are members of support groups in Kirinyaga. To reduce contamination, the unit of randomization will be the support group. Peer supporters in the intervention arm will receive training to deliver the intervention. The intervention will include monthly group education on DR and individual member reminders to take the eye examination. The effectiveness of this intervention plus usual care will be compared to usual care practices alone. Participant data will be collected at baseline. The primary outcome is the proportion of PLWD who take up the eye examination at six months. Secondary outcomes include the characteristics of participants and peer supporters associated with uptake of eye examination for DR. Intention-to-treat analysis will be used to evaluate the primary and secondary outcomes.

**Discussion:**

Eye care programs need evidence of the effectiveness of peer supporter-led health education to improve attendance to retinal screening for the early detection of DR in an African setting. Given that the intervention combines standardization and flexibility, it has the potential to be adopted in other settings and to inform policies to promote DR screening.

**Trial registration:**

Pan African Clinical Trial Registry PACTR201707002430195, registered 25 July 2017, www.pactr.org

## Background

The global prevalence of diabetes has escalated in recent decades, with important implications on the health system. In 2015 the International Diabetes Federation estimated that there were 415 million people with diabetes aged 20–79 years (global prevalence of 8.8%), and this is predicted to increase to 642 million by 2040 (global prevalence of 10.4%). [1] The incidence and prevalence of diabetes is increasing disproportionately faster in resource-poor regions and 75% of people living with diabetes currently reside in low- and middle-income countries. [1–3] This dramatic increase in incidence is occurring in both rural and urban areas. [4] The regional prevalence for Africa was 3.8% in 2015, and the number of people with diabetes in this continent is expected to increase by 140% between 2015 and 2040. [1] In Kenya, the STEPwise survey for risk factors of non-communicable diseases in 2015 found a diabetes prevalence of 2% in the population 18–69 years, and 5.4% in the population 45–59 years. [5]

All patients with diabetes are at risk of developing diabetic retinopathy (DR), the most severe and progressive ocular complication of diabetes. One third of patients with Type 2 diabetes have DR while 10% of them have sight-threatening DR, which represents a significant public health concern. [6, 7]. A population-based study in Nakuru county, Kenya found that 35.9% of people living with diabetes (PLWD) have DR [8]. Visual impairment and blindness from DR is preventable mainly through early detection and timely treatment. Since DR is asymptomatic until the advanced stages, regular retinal screening is of paramount importance. DR meets the Wilson and Jungner (1968) criteria for screening, and current clinical guidelines support annual screening [9–12]. Participation of PLWD in regular retinal screening, has been shown to be clinically effective in preventing blindness and is also cost-effective. [13, 14]

In developed countries, health systems have formal surveillance programmes for detection of DR. Kenya does not have a national population-based DR screening service where PLWD are systematically invited for screening, but opportunistic screening is available in various hospitals. Importantly, participation and re-participation rates in screening for DR are sub-optimal in Kenya and other resource poor settings. [15–22]. The determinants of the attendance to retinal examination are complex and include both supply and demand factors. [23, 24] For instance, a Tanzanian study found that PLWD also have limited awareness on diabetic retinopathy, particularly on the need for annual eye examination [25]. This is a barrier that appropriate demand side interventions could address. A health system assessment conducted before this study has shown that 87% of PLWD in Kenya have an unmet need for *annual* retinal screening. [22] One of the gaps associated with this is the lack of strong links between diabetes services and eye care services. There is need for context-specific pragmatic solutions to address this gap.

A systematic review of interventions to increase diabetic retinopathy screening attendance reported that several strategies are effective, including those targeting the patient (e.g. increasing patient awareness), the health care practitioner (e.g. improving adherence to recommendations) or the organization (e.g. improving patient records) [26]. Members of diabetes support groups (DSGs) are a population subgroup that might benefit from additional support to initiate screening, and adhere to re-screening. Targeting screening interventions towards PLWD in DSGs provides a timely opportunity for three reasons. Firstly this is a community resource that is already available in the community setting. Secondly this population is likely to consider health as an important rationale for behaviour change and the health seeking behaviour of members is potentially malleable to change through peer support. Peer support refers to the provision of emotional and informational support from a created social network member who is considered an equal and who has characteristics similar to the target population. [27] Thirdly it is an economical, culturally-sensitive and flexible intervention for improving diabetes care and outcomes. [28, 29]

**Fig. 1:**
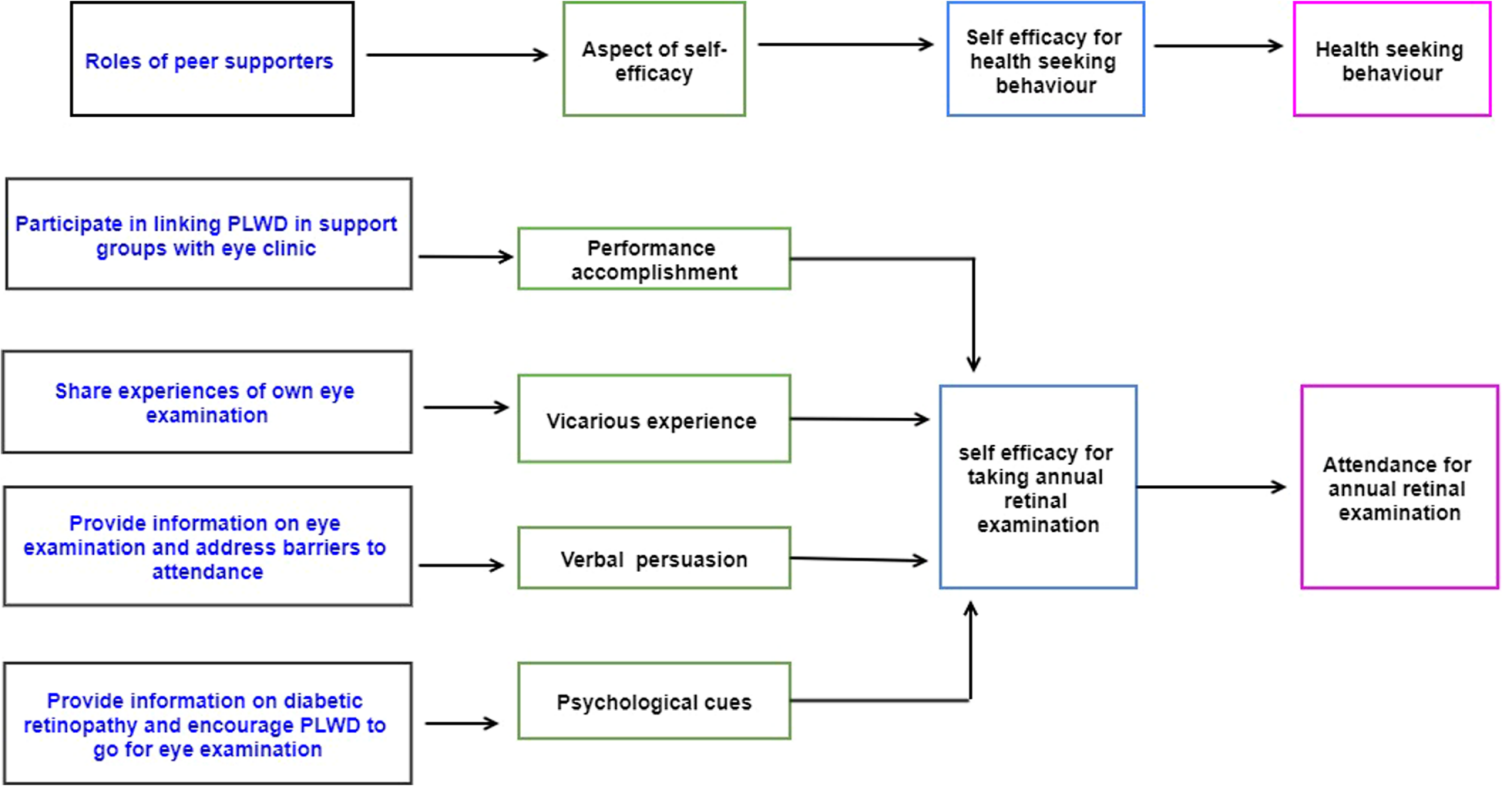
Peer support and self-efficacy for retinal examination

Self-efficacy is a direct predictor of health behaviour, according to the social cognitive theory and the self-efficacy theory [30] [31]. Self-efficacy is a predictor of uptake of screening for DR among PLWD. [32] Interventions that improve patients’ self-efficacy decrease perceived barriers and improve the likelihood of initiating the desired health behaviour. There are four main sources of self-efficacy [31]: (i) Successful performance accomplishments (e.g. having attended a previous eye examination) (ii) vicarious experience (e.g. learning that peers have successfully attended an eye examination) (iii) verbal persuasion (e.g. encouragement and recommendation to go for an eye examination by a trusted person, such as a peer or a health care worker) and (iv) psychological cues (decreased sense of isolation of PLWD interacting with a peer, or increased awareness of the risk of DR after receiving educational messages on DR). Fig. 1 shows how self-efficacy for taking a retinal examination might improve through peer support in the Uptake of Retinal Examination in Diabetes (DURE) study.

### Rationale

Although there is evidence that peer support improves glycaemic control and quality of life among adult PLWD, and that peer support is both cost-effective and flexible [33], evidence on whether or not it would increase uptake of eye examination in an agrarian African population is lacking. DURE study aims to provide this evidence.

The development of the intervention has been informed by the following:

i. A review of the literature on peer support in diabetes [34–42] and other chronic conditions in resource poor settings. [43–51] There is evidence that peer approach is widely used in the management of diabetes, to promote  physical activity, healthy eating and improvemt in glycaemic control.
ii. The results of a recent published meta-analysis of randomized clinical trials on effectiveness of peer-support for glycaemic control in Type 2 diabetes [52] which concluded that peer support had a significant impact on improving HbA_1c_ levels in patients with poor glycaemic control.
iii. A health system assessment in three counties of Kenya conducted before this study showed that services for DR are underutilised: 74% of PLWD have *never* had a retinal examination in their lifetime, and 76% have *never* had a recommendation for an eye examination by their diabetes care provider [22].
iv. Evidence that improving health literacy, provider patient interaction and linking patients to health care improves patients’ self-efficacy and glycaemic control. [42, 53, 54]

### Aim

To evaluate, by means of a pragmatic cluster randomized controlled trial, the effectiveness of a peer supporter- led community education programme in Kirinyaga county, Kenya.

### Research questions

1. To what extent can health education delivered by peer supporters increase the demand for annual retinal examination among PLWD?
2. What are the contextual factors that determine the effectiveness of the intervention?

### Hypothesis

The hypothesis is that the proportion of PLWD having a retinal examination for DR is higher in diabetes support groups (DSGs) allocated to the peer supporter-led educational package than in DSGs randomized to the usual standard of care.

## Methods

### Design

This is a two-arm pragmatic cluster randomized controlled trial with additional process evaluation. It is a complex intervention to empower patients to undergo an annual eye examination. It is complex because those delivering and receiving the intervention require to demonstrate different behaviours and to engage in multiple interactions. [55] Its design is guided by the Medical Research Council framework for complex interventions, available at https://mrc.ukri.org/documents/pdf/complex-interventions-guidance/ [55].

The study will be conducted in accordance with the Consolidated Standards of Reporting Trials (CONSORT) 2010 statement and its extension to cluster randomized clinical trials (cRCTs). [56, 57] The cRCT design is adopted for the following reasons: (i) to reduce the effect of intervention contamination, as compared to an individually randomised trial, as patients in the same DSG often interact with one another (ii)to make it feasible to study the effect of the intervention at the individual level and the cluster level.

### Definition of eye examination for DR

We define this test as: measurement of visual acuity and a retinal examination through a dilated pupil conducted by an eye care worker (using either an ophthalmoscope, a slit lamp or a retinal camera). Retinal examination for DR and DR screening in this protocol are used interchangeably.

### Study setting

This trial will be conducted among the DSGs in Kirinyaga county, Kenya. The target population is members of the 16 support groups and volunteer peer supporters within these groups. Eye examination will be conducted at Kerugoya County Referral Hospital.

### Sample size calculation

We aim to randomize seven diabetes patient support groups (clusters) with an average membership of 50 each to each arm. The study thus has two arms of equal size (350 participants in each arm).This sample size has been calculated using standard formula for sample size for cRCTs and taking into consideration the primary outcome of interest [58, 59]. A 15% loss to follow-up contingency has been built into the sample size calculation. This sample size would have 80% power to detect a two-fold difference in the proportion of PLWD who take up eye exam, with a 5% level of significance. Member registers of the DSGs will be obtained from the team lead. These registers will be the frame for identification of participants for the study.

### Pilot study

A pilot study will be conducted in two clusters with 50 PLWD in each arm (intervention arm and control arm), selected through convenience sampling. The pilot will be conducted for 3 months and will involve: Testing study operational procedures; Implementation of the intervention in the intervention clusters; Testing study instruments for quantitative and qualitative data collection (questionnaires, observation sheets and topic guides); Outcomes evaluation. The primary outcome will be the proportion of participants in each arm that take up eye examination.

### Inclusion and exclusion criteria

Participants will be included if they are PLWD aged 18+ years, will reside in Kirinyaga for the next 12 months, are members of DSGs in Kirinyaga, have a mobile phone and are willing to participate in the study. In addition to these criteria, peer supporters will be selected from those willing to participate as peer supporters, willing to commit two days for training and many hours of peer support, fluent in Kikuyu or Kiswahili, and have had a retinal examination for DR before the start of the study. PLWD who will be excluded are those already attending annual retinal screening, have a severe debilitating medical condition, are already on treatment for DR or do not meet the inclusion criteria.

### Recruitment

**Fig. 2:**
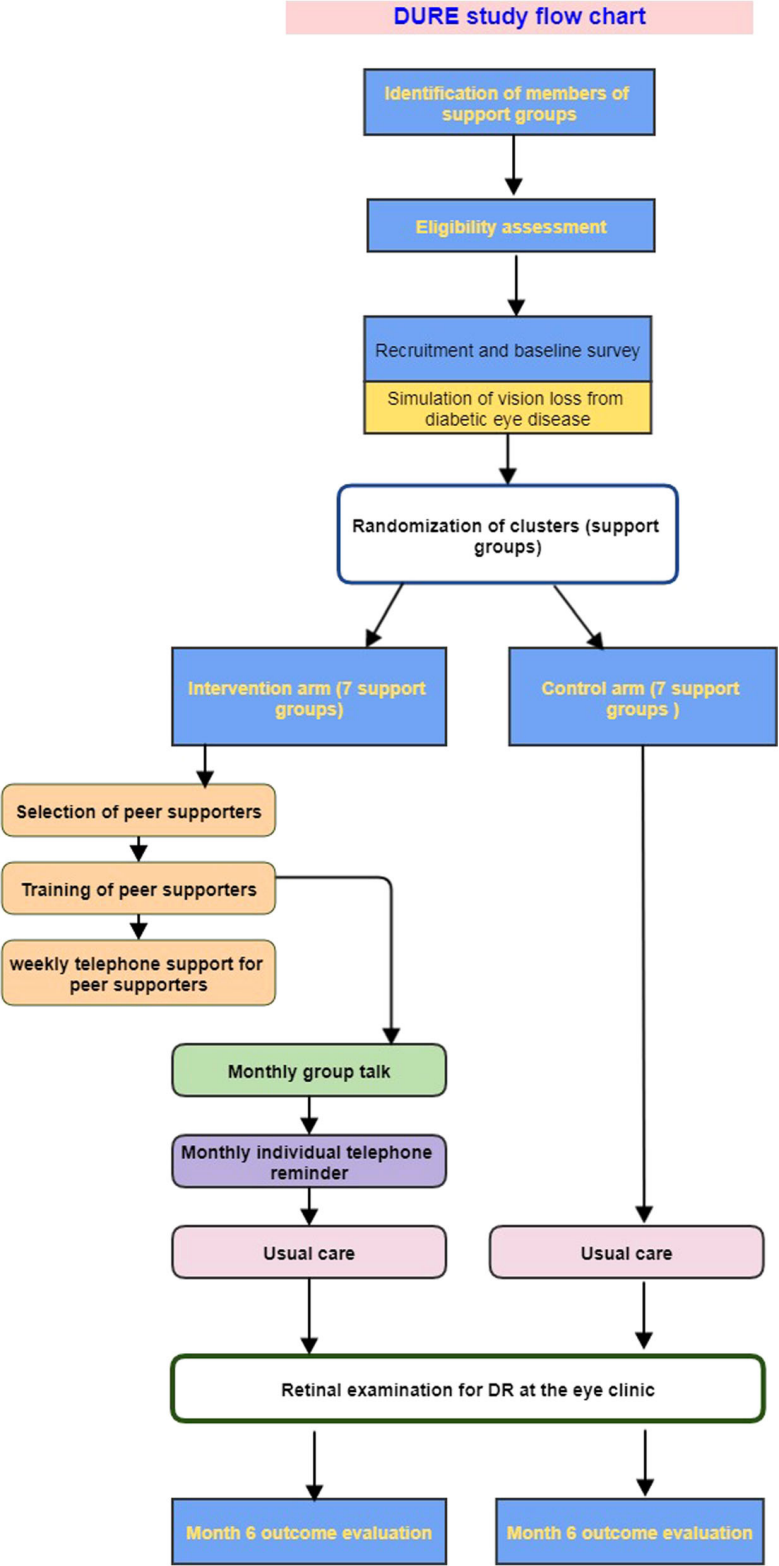
Flow diagram for the trial

Eligible participants will be recruited into the study by the research nurse, who will also obtain informed consent at the cluster level using the consent form approved for the study. Participants will be asked for consent to receive the intervent ion and for follow up. If the patient does not consent, reasons will be sought and recorded. After recruitment, a unique identifier number will be issued. All those recruited will be given an identification card which contains the name and a unique study number. They will be required to present this card at the eye clinic when they go for retinal examination. The flow diagram for the study is presented in Fig. 2.

### Randomization

Randomization will be done after recruitment. The randomization will be through computer generated random numbers prepared by a statistician (DM) using STATA version 15 (StataCorp 2017), away from the project site. The allocation sequence will be concealed from the other trial personnel. Block randomization with block sizes of two or four will be used to ensure that the two arms are balanced over time, and to maintain unpredictability of allocation. Masking will not be possible but only the research team will have formal knowledge of the allocation.

### Intervention

Two peer supporters will be recruited from each cluster in the intervention arm (one male and one female). These peer supporters will be selected from volunteers who meet the specified criteria. They will receive structured training in a two-day workshop. The content of the training sessions includes: an introduction to the project, the role of the peer supporter, diabetic eye disease and DR, retinal examination for DR, communication skills, managing groups, confidentiality and behaviour change. The training team will include a certified diabetes educator.

They will receive support to retain them in the study (airtime vouchers for delivering the telephone reminders). They will also receive a weekly telephone call from the principal investigator for support. The intervention group will receive the usual care, a monthly group talk and a monthly individual telephone reminder to attend retinal examination. The key messages to be delivered in the group talk are shown in Table 1. The control group will receive the usual standard of care, which consists of ad hoc diabetes educational talks, blood sugar and blood pressure measurements during support group meetings.

**Table 1:** Key messages to be delivered to participants

*Messages on diabetic eye disease*	
1	Diabetes causes several complications in the eye, including DR
2	DR is a progressive condition that leads to blindness if treatment is not provided in good time
3	DR has no symptoms until the advance stages
4	An eye check by an eye specialist can detect damage to the eyes before symptoms develop
5	All persons with diabetes should have their eyes checked once every year by an eye specialist, even before any symptom or poor vision develops
6	Do not wait for your vision to get worse or for any other symptom to occur before you see an eye specialist
7	If eyes are found to be normal at your eye check by an eye specialist, please continue with an eye check annually
8	If you notice any abnormality with your eyes between your clinic appointments, visit the eye specialist as soon as possible. It may not mean that you have diabetic eye disease, it may be a simple problem that requires treatment.
9	The eye check may help to determine if your sugar, blood pressure, and lipid control needs to be re-assessed. Good control of your blood sugar levels, blood pressure and cholesterol reduces the risk of diabetes-related sight loss.
10	If you are found to have DR the eye specialist will inform you about the diagnosis, and how it will be treated.
*Messages on retinal examination for DR*	
1.	Ensure you have a dilated eye examination at the eye clinic at least once a year.
2.	You do not need to have a referral note to go to the clinic. However we will give you a card to present at the clinic.
3.	At the eye clinic, your vision will be checked first.
4.	A examination for DR is different from any other type of eye examination. It is called a dilated eye examination.
5.	In this examination, the doctor puts eye drops into your eyes to dilate (widen) your pupils. This allows the doctor to have a good view of the back of the eye. Both eyes need to be examined.
6.	The examination is not painful. When the eye drops are first instilled, there may be a slight stinging sensation but this only lasts about a minute. You may feel uncomfortable because of light sensitivity and blurred vision once the pupils are dilated.
7.	Do not be afraid to ask the doctor questions about the examination or about diabetic eye disease.

### Data collection

Standardized operating procedures will be used to collect data at baseline, using approved tools in the study proposal. Demographic and anthropometric (height, weight, waist circumference) as well as blood pressure, blood sugar and visual acuity will be recorded at baseline. Study participants will be given their body mass index and blood pressure measurements in the field, at the point of data collection; where these results are abnormal, participants will be referred to a health worker. Completed questionnaires will be monitored and data entry staff will be trained to minimize errors in data entry into computerized databases. Identifiers will be removed from participant data, and all paper data will be stored in locked cabinets. Electronic data will be password protected for confidentiality. A detailed data management plan is included in the study proposal.

### Follow up

Participants in both arms will be followed up for six months to assess attendance to retinal examination. Participants who are lost to follow up will be identified at the monthly contact points with peer supporters. Three home visits will be made to trace participants who are lost to follow up. Characteristics of those lost to follow-up and reasons for loss will be evaluated.

At the end of six months two separate focus group discussions will be held with two participants from each intervention cluster in each arm (*n* = 14 for each focus group discussion) to explore the experience of the support groups with the intervention. A focus group discussion will also be held with peer supporters to explore the impact of ‘peer supporting’ on the management of their own diabetes, and their role in the health care team.

### Process evaluation

A process evaluation will be conducted using qualitative interviews and non-participant observation (Table 2). The findings of the process evaluation will be evidence on why and how the intervention worked. The following domains of the intervention will be evaluated:

1. Whether the intervention activities are implemented as planned (fidelity).
2. The extent to which the intervention reaches the PLWD (reach).
3. The degree to which PLWD are exposed to the intervention package (dose).
4. The extent to which the intervention is acceptable to PLWD and to eye care workers (acceptability).

5) The contextual factors that may have an influence on the theory of change (context).

**Table 2:** Domains and methods for process evaluation

Source	Domain	Data collection method	Stage of the trial
Trial registers	Recruitment Retention	Registers in the trial office	Throughout the trial (*n* = 700)
Participants	Fidelity Reach Dose received Effectiveness	Participant Questionnaire 2 Focus group discussions at 6 months	At recruitment (n = 700) Three months: n = 10% pf participants in each intervention cluster(35) Six months: *n* = 10-% of participants in each cluster (35) *N* = 28
Non-participant observations by PI	Recruitment Fidelity Dose delivered Context	PI Field notes	N = 2 group meetings per intervention cluster during the trial (14)
Peer supporters (PS)	Effectiveness Reach Fidelity Dose delivered Context	PS Questionnaires PS Diary for telephone calls PS Group session report form Focus group discussion at 6 months	After training (n = 14) Through the trial (n = 14) Throughout the study (1 report form per group meeting per PS) N = 7
Eye care workers Key informants	Context	In-depth interviews	At 6 months *N* = 3 *N* = 7
Research project manager	Reach Fidelity Dose delivered	Reports	At recruitment of PS At training the PS At 3 and 6 months
Research nurse	Outcome evaluation procedures	Report	At 3 months and 6 months
Study steering committee	Context	Spreadsheet of external events that may have affected study outcomes	At 6 months

*PI* Principal Investigator, *PS* Peer supporter

### Assessment of outcome

#### Primary outcome

Rates of eye examination in each arm will be assessed in each arm at the end of six months. This outcome will be assessed by an independent and masked research nurse who will review the eye clinic records of all participants. The outcome will be recorded on the outcome evaluation form for each participant. The form contains identification details of each participant recruited into the study (name, residence, telephone number) and will thus differentiate them from other patients who are examined in the eye clinic. The form does not contain information on the intervention arm to which the patient is allocated. The project manager will receive the completed outcome evaluation forms and link the data to the participant database for each arm.

#### Secondary outcomes

These outcomes will be assessed at six months:

1. Contextual factors that affect the effectiveness of the intervention
2. Characteristics of peer supporters associated with uptake of eye examination
3. Barriers to uptake of eye examination among PLWD.

These outcomes will be evaluated using the database for participants and peer supporters, as well as data from focus group discussions with peer supporters and in-depth interviews with eye care workers at six months.

### Statistical analysis

Baseline comparability of the two groups will be assessed to check that the important confounders and baseline characteristics that would affect uptake of eye examination are balanced between the two arms through randomisation. If the arms are found to be substantially imbalanced an appropriately adjusted logistic regression model will be used.

Study-wide pooled analysis will be conducted for the primary outcome. Missing data will be reported using standard flow charts. Repeated measures mixed models regression with adjustment for age, sex, and baseline anthropometric measures will be used to compare the two groups for the primary outcome.

Analysis will be conducted on intention-to-treat basis. Regression analysis will be used to determine the extent to which individual and support group characteristics are associated with the primary outcome. Models for comparing continuous outcomes will use linear regression while models for categorical outcomes will use logistic regression. Kaplan-Meier analysis will be used to plot the survival curves for both treatment arms. Cox regression will be used to assess the impact of the intervention on time to first eye examination. The hazard ratio will be estimated with Cox regression, adjusting for substantial baseline imbalances if appropriate. Interim analysis is not planned.

### Data monitoring

The principal investigator will coordinate and monitor all recruitment, intervention and follow up procedures. A data monitoring committee will not be required. There is no reason to expect significant adverse effects and there are no stopping rules. The principal investigator will have access to all the trial data sets.

### Harms

Neither arm of the trial has serious anticipated harms. The retinal examination involves the use of mydriatic eye drops. This may cause temporary blurring of vision, but this is only expected to last for a few minutes or hours. In this trial the drugs will be instilled by highly experienced clinicians, and patients will be made aware of this effect beforehand. Any unexpected effects of the trial will be documented and reported to the sponsor and ethics committees.

### Dissemination

The dissemination strategy will include a summary of the findings for support groups, a report to Kerugoya County government and the Ministry of Health Kenya, publications in peer-reviewed journals and presentations at national and international conferences.

### Post-trial care

It is recommended that all PLWD have an annual retinal examination for DR, and more frequent examinations are required for those found to have any stage of DR. This is best practice that is recommended by the national guidelines for screening and management of DR in Kenya. [12] The service will continue to be available as routine care to PLWD at the Kerugoya County Referral Hospital beyond the study.

## Discussion

This study is pragmatic in that it tests the effectiveness of this intervention in the real-world situation of the community and the health system in Kirinyaga. There is a strong need to develop interventions that can reach PLWD populations in real world settings to ensure that any effect found is generalizable.

Public health strategies to manage the diabetes and DR in sub-Saharan Africa (SSA) are known to be inadequate or non-existent. [60, 61] Given that we are at the emergence of the epidemic, this is an appropriate time to develop contextual interventions that will enable our health system to cope with this challenge. To our knowledge, this is the first study that has targeted the DSG population in DR research. The use of peer support in DR is a relatively new field and little has yet been published on the topic.

The trial is important for a number of reasons. For the *individuals with diabetes*, this trial is in line with the growing global focus on patient empowerment. The PLWD will be empowered to demand for retinal examination, thus reducing demand side barriers to uptake of the examination. These actively engaged PLWD will be linked to eye care providers by the peer supporters. The *Chronic Care Model*, which has been proposed as a suitable model for managing diabetes, emphasises on the need to implement such links between patients and the health system using community resources. [62] [47] As all PLWD are at risk of DR, empowerment to initiate and maintain screening will be beneficial to all.

For the *support groups*, if this intervention positively influences uptake of retinal examination, this could in turn influence how the DSGs define their role. It has potential to instigate a new agenda, making the groups key sites for preventive public health initiatives that are adaptable, feasible and embedded within support group culture. The peer supporters will remain a valuable resource in the DSG, which enhances sustainability of effect.

For *Kirinyaga county*, our study findings might help the county (formerly district) health services to develop initiatives to promote early detection of DR, by involving DSGs, empowering patients and developing effective referral systems for DR services. The role the support groups can play in strengthening the health system for diabetic retinopathy in the county will become explicit.

The intervention will be provided by trained peer supporters, which is a form of task-shifting. Task shifting is commonly applied in both diabetes and eye care services in our setting. It helps to address the severe shortage of human resources for health. The peer supporters will refer patients to the eye clinic, thus linking diabetes patients with eye clinics and strengthening the referral system in the county.

In *national context*, Kenya aims to achieve universal eye health, which includes care for DR. This study provides a framework for the promotion of retinal screening in the population with the risk of developing DR. If effective, the intervention would be a sustainable and scalable to other countries.

In the *international contexts*, the DURE study has the potential to extend current evidence and inform the scientific debate as to whether embedding retinal screening into DSGs is an effective next step toward meeting health goals.

The explicit use of a theoretical construct (self-efficacy theory) to conceptualise the potential determinants that would influence attendance to DR screening is a key strength of the study. It enhances the understanding of the plausibility of the intervention. The intervention package combines both standardization and flexibility, which allows for scalability in diverse settings. A further strength of this study is the inclusion of process evaluation, which will assist in the interpretation of how and why the intervention did, or did not, bring about the predicted effects.

The study has potential limitations. There are only 16 support groups in the county, which limits the possibility of increasing the number of clusters to further enhance statistical power. Delayed recruitment of the required sample size and loss to follow-up during the trial may be a challenge. In mitigation, a 15% loss to follow-up contingency has been built into the sample size calculation. Sample attrition can result from any inaccuracies in the data collection, such as incorrect address and telephone number information. Other diabetes studies have documented that patients were unable or unwilling to participate due to transportation issues and lack of time or interest [63]. However this is not anticipated because: alternative contact information of participants will be documented, only one visit to the eye clinic is required of participants, and the intervention is expected to build participants’ self-efficacy. Attrition bias may occur if whole clusters drop out, however this is not anticipated as the study period is short.

Despite these limitations, DURE study illustrates the tremendous potential of implementing pragmatic cluster RCTs in the diabetes support group setting. Implementing the trial in this at-risk population will be an invaluable learning opportunity. Many of the lessons learned from this experience could be useful to other research projects.

### Trial status

At the time of submission, the trial is at the stage of enrolment.

## Abbreviations

CONSORT: Consolidated Standards of Reporting Trials
cRCT: Cluster Randomized Clinical Trial
DR: Diabetic Retinopathy
DSG: Diabetes support group
DURE: Uptake of Retinal Examination in Diabetes
PACTR: Pan African Clinical Trials Registry
PLWD: People living with diabetes
SE: Self-Efficacy
STDR: Sight-threatening diabetic retinopathy
